# Seroprevalence of Dengue and Chikungunya Virus Antibodies, French Polynesia, 2014–2015

**DOI:** 10.3201/eid2403.171149

**Published:** 2018-03

**Authors:** Maite Aubry, Anita Teissier, Michael Huart, Sébastien Merceron, Jessica Vanhomwegen, Mihiau Mapotoeke, Teheipuaura Mariteragi-Helle, Claudine Roche, Anne-Laure Vial, Sylvianne Teururai, Sébastien Sicard, Sylvie Paulous, Philippe Desprès, Jean-Claude Manuguerra, Henri-Pierre Mallet, Allison Imrie, Didier Musso, Xavier Deparis, Van-Mai Cao-Lormeau

**Affiliations:** Aix Marseille University, IRD, AP-HM, SSA, VITROME, IHU-Méditerranée Infection, Marseille, France (M. Aubry, A. Teissier, T. Mariteragi-Helle, S. Teururai, D. Musso, V.-M. Cao-Lormeau);; Institut Louis Malardé, Papeete, French Polynesia (M. Aubry, A. Teissier, M. Mapotoeke, T. Mariteragi-Helle, C. Roche, S. Teururai, D. Musso, V.-M. Cao-Lormeau);; Centre d’Épidémiologie et de Santé Publique des Armées, Marseille, France, and Unité Mixte de Recherche Sciences Economiques et Sociales de la Santé et Traitement de l'Information Médicale, Marseille (M. Huart, S. Sicard, X. Deparis);; Institut de la Statistique de la Polynésie Française, Papeete, and Institut National de la Statistique et des Études Économiques, Sainte Clotilde, La Réunion (S. Merceron);; Institut Pasteur, Paris, France (J. Vanhomwegen, S. Paulous, J.-C. Manuguerra);; Direction de la Santé de la Polynésie Française, Papeete (M. Mapotoeke, A.-L. Vial, H.-P. Mallet);; Direction Départementale de la Cohésion Sociale et de la Protection des Populations, Yonne, France (A.-L. Vial);; Université de la Réunion, Sainte Clotilde (P. Desprès);; University of Western Australia, Perth, Western Australia, Australia (A. Imrie)

**Keywords:** seroprevalence, immunoglobulin G, arboviruses, dengue viruses, chikungunya virus, Zika virus, French Polynesia, Pacific, viruses, IgG

## Abstract

We investigated dengue and chikungunya virus antibody seroprevalence in French Polynesia during 2014–2015. Dengue virus seroprevalence was ≈60% among schoolchildren and >83% among the general population; chikungunya virus seroprevalence was <3% before and 76% after Zika virus emergence (2013). Dengue virus herd immunity may affect Zika virus infection and pathogenesis.

In French Polynesia, the only recognized actively circulating arboviruses were the 4 dengue viruses (DENV; family *Flaviviridae,* genus *Flavivirus*) (*1*,*2*) until Zika virus (family *Flaviviridae,* genus *Flavivirus*) emerged there in 2013 (*3*), followed by chikungunya virus (family *Togaviridae*, genus *Alphavirus*) in 2014 (*4*). Serosurveys conducted among blood donors in French Polynesia during 2011–2013, before these outbreaks, confirmed the absence of Zika and chikungunya virus circulation and assessed DENV antibody seroprevalence at ≈80% at that time (*5*,*6*). Another study conducted after the emergence of Zika virus showed Zika virus antibody seroprevalence rates ± 95% CIs to be 49% ± 7% among the general population and 66% ± 5% among schoolchildren in 2014 and 22% ± 6% among the general population in 2015 (*7*). We report seroprevalence of antibodies against the 4 DENVs and chikungunya virus in French Polynesia in 2014–2015 and discuss the possible role of anti-DENV herd immunity on Zika virus infection and pathogenesis.

## The Study

To assess antibody seroprevalence against DENV types 1–4 and chikungunya virus in the population of French Polynesia, we conducted 3 cluster samplings (*7*). During February–March 2014, we sampled 196 participants from the general population of the 8 most inhabited islands of the 5 French Polynesia archipelagos: Tahiti and Moorea (Society), Rangiroa and Makemo (Tuamotu), Nuku Hiva and Hiva Oa (Marquesas), Rurutu (Australs), and Rikitea (Gambier). To better estimate seroprevalence of antibodies against these viruses among children, during May–June 2014, we recruited 476 schoolchildren from primary and high schools on the most populous island (Tahiti). To increase accuracy of seroprevalence data for DENVs and assess postoutbreak chikungunya virus seroprevalence, during September–November 2015, we sampled 700 members of the general population from the most inhabited archipelago (Society). All participants were asked whether they had had symptoms suggestive of past dengue disease. Because chikungunya virus emerged in French Polynesia in late 2014, symptoms suggestive of past disease were recorded only from participants sampled in 2015. The study was conducted in accordance with the French Polynesia Ethics Committee (agreement 60/CEPF_06/27/2013).

We performed detection of DENV and chikungunya virus IgG on blood samples collected in 2014 by using a recombinant-antigen–based indirect ELISA (*5*,*6*) and tested samples collected in 2015 by microsphere immunoassay (MIA) (*8*) using the same recombinant antigens as for the ELISA. Among the samples collected in 2015, we selected 20 to be a representative panel of the different antibody profiles found by MIA and tested them for neutralizing antibodies against each dengue, chikungunya, and Zika virus (*8*). We analyzed data by using GraphPad Prism version 6.03 software (https://www.graphpad.com/) and the Fisher exact test. We set significance at p<0.05.

Overall seropositivity rates for antibodies against >1 DENV were 96% ± 3% among the general population and 60% ± 5% among schoolchildren in 2014 and 83% ± 3% among the general population in 2015 (Table 1). Seroprevalence of DENV antibodies did not differ significantly between archipelagos, except for DENV-3, which differed between Society (76% ± 18%) and Austral-Gambier (53% ± 15%) Islands (p = 0.034). In all 3 groups of participants, we found the highest seropositivity rates for DENV-1 antibodies and the lowest for DENV-2. Seropositivity rates in 2014 and 2015 for all DENV antibodies were significantly lower for schoolchildren (median age 11 years) than for the general population (median age 41 years in 2014 and 43 years in 2015) (all p<0.0001). Seroprevalence of chikungunya virus antibodies was 3% ± 3% among the general population and 1% ± 1% among schoolchildren in 2014 and 76% ± 5% among the general population in 2015 after the outbreak.

**Table 1:** Seropositivity for antibodies against dengue and chikungunya viruses among participants randomly recruited, French Polynesia, 2014–2015*

Virus	Seropositivity, no. (% ± 95% CI)

General population, Feb–Mar 2014					Schoolchildren, May–Jun 2014, Society, n = 476	General population, Sep–Nov 2015, Society, n = 700
Society, n = 49	Tuamotu, n = 49	Marquesas, n = 49	Austral –Gambier, n = 49	Total, n = 196
Dengue							
>1 type	46 (94 ± 8)	49 (100 ± 0)	46 (94 ± 8)	47 (96 ± 6)	188 (96 ± 3)	285 (60 ± 5)	582 (83 ± 3)
Type 1	42 (86 ± 16)	47 (96 ± 5)	41 (84 ± 13)	42 (86 ± 11)	172 (88 ± 6)	239 (50 ± 5)	562 (80 ± 4)
Type 2	23 (47 ± 17)	22 (45 ± 13)	26 (53 ± 12)	28 (57 ± 12)	99 (51 ± 7)	0	127 (18 ± 4)
Type 3	37 (76 ± 18)	33 (67 ± 21)	35 (71 ± 14)	26 (53 ± 15)	131 (67 ± 9)	72 (15 ± 3)	384 (55 ± 4)
Type 4	31 (63 ± 8)	32 (65 ± 21)	29 (59 ± 15)	27 (55 ± 15)	119 (61 ± 8)	69 (14 ± 3)	293 (42 ± 7)
Asymptomatic	36/46 (78 ± 12)	29/49 (59 ± 14)	24/46 (52 ± 14)	34/47 (72 ± 13)	123/188 (65 ± 7)	230/285 (81 ± 5)	299/582 (51 ± 4)
Chikungunya	3 (6 ± 8)	1 (2 ± 4)	1 (2 ± 4)	1 (2 ± 4)	6 (3 ± 3)	2 (1 ± 1)	529 (76 ± 5)
Asymptomatic	ND	ND	ND	ND	ND	ND	70/529 (13 ± 2)

*95% CIs were calculated by taking into account the cluster sampling design and using the Fisher exact test. Median ages of participants among the general population, 38–47 y; of schoolchildren, 11 y. ND, not determined.

According to information provided by a questionnaire, the percentages of asymptomatic DENV infections were 65% ± 7% among the general population and 81% ± 5% among schoolchildren in 2014 and 51% ± 4% among the general population in 2015. The percentage of asymptomatic chikungunya virus infections in participants recruited in 2015 was 13% ± 2%. Among the 20 samples positive by MIA for IgG against DENV-1–4 and chikungunya virus in this study and against Zika virus in the previous serosurvey (*7*), the proportions showing neutralizing activity were 8/9 (89%) for DENV-1, 5/5 (100%) for DENV-2, 6/6 (100%) for DENV-3, 6/6 (100%) for DENV-4, and 7/8 (88%) each for chikungunya and Zika viruses (Table 2).

**Table 2:** Results of microsphere immunoassay and neutralization assay for dengue, chikungunya, and Zika viruses for 20 participants sampled from the Society archipelago, French Polynesia, September–November, 2015*

Participant ID	Years in French Polynesia	Virus																
		Dengue 1			Dengue 2			Dengue 3			Dengue 4			Chikungunya			Zika‡	
MIA	NTA†	MIA	NTA	MIA	NTA	MIA	NTA	MIA	NTA	MIA‡	NTA
Pueu-6	1	–	<10		–	<10		–	<10		–	<10		+	80		–	<10
Papeete-62	2	–	<10		–	<10		–	<10		–	<10		–	<10		–	10
Papeete-63	2	–	<10		–	<10		–	<10		–	<10		–	<10		–	40
Papeete-35	6	–	<10		–	<10		–	<10		–	<10		–	<10		–	20
Punaauia-96	6	–	<10		–	<10		–	<10		–	<10		–	<10		–	10
Pirae-6	10	–	<10		–	<10		–	<10		–	80		–	<10		+	160
Mahina-26	13	–	<10		–	<10		–	<10		–	<10		+	320		+	160
Punaauia-61	15	–	640		–	<10		–	<10		–	<10		–	<10		–	<10
Faaa-54	17	+	10		–	<10		–	<10		–	<10		+	160		+	>1,280
Toahotu-1	18	–	<10		–	<10		–	<10		+	80		–	<10		–	<10
Punaauia-93	21	–	<10		–	640		–	<10		–	<10		–	<10		–	20
Punaauia-36	24	–	40		–	640		–	40		–	80		+	160		–	20
Papenoo-10	28	+	640		+	>1,280		+	>1,280		+	640		–	<10		–	<10
Moorea-59	36	+	320		–	<10		+	>1,280		+	>1,280		–	<10		+	640
Paea-48	38	+	160		–	<10		–	80		–	80		–	<10		+	160
Papeari-17	44	+	320		+	>1,280		+	80		+	160		+	20		+	320
Papenoo-1	49	+	160		+	>1,280		+	20		+	80		+	640		+	<10
Faaa-22	54	+	80		+	640		–	320		–	160		+	40		–	<10
Papeete-10	55	+	320		+	>1,280		+	80		+	640		+	<10		+	160
Afaahiti-7	67	+	80		–	640		+	320		–	160		–	<10		–	<10

*ID, identification; MIA, microsphere immunoassay; NTA, neutralization assay; +, positive; –, negative. †Neutralization activity was considered positive for serum samples with a 50% neutralization antibody titer >20. ‡Results from Aubry et al. (7).

## Conclusions

The finding of DENV IgG in >80% of the general population sampled in 2014 and 2015 corroborates past high transmission rates for these viruses in French Polynesia (*1*,*2*,*6*,*9*). The lower seropositivity rates for antibodies against DENVs among children is consistent with seroprevalence rates for antibodies against viruses endemic to the region, which are expected to increase with duration of exposure (*6*). Finding the highest seroprevalence rate against DENV-1 in the general population and schoolchildren is consistent with the long-term circulation of this virus in French Polynesia during 2001–2009 and since 2013 (*1*,*9*). DENV-2 has not circulated in French Polynesia since 2000 (*9*), which is consistent with the lowest seroprevalence rate against this virus. The reduced population immunity against DENV-2 coupled with the circulation of this virus in neighboring Pacific Islands and detection of imported infections in travelers entering French Polynesia from Vanuatu in February 2017 (*9*) indicates that French Polynesia is at risk for a new epidemic. Although seroprevalence rates for antibodies against DENVs are consistent with epidemiologic data, bias associated with the presence of cross-reactive antibodies produced during a previous flavivirus infection could have resulted in detection of antibodies against viruses to which the persons were never exposed. The proportion of asymptomatic DENV infections assessed in this study seems to be consistent with reported rates (*10*). However, because the questionnaire asked about clinical manifestations suggestive of past DENV infection over a lifetime or during time spent in French Polynesia, the estimates may be strongly limited by recall bias.

The low seroprevalence rates for chikungunya virus antibodies for the general population (3% ± 3%) and schoolchildren (1% ± 1%) in 2014 corroborate previous findings for blood donors sampled during 2011–2013 (3%) (*5*), suggesting that this virus did not actively circulate in French Polynesia before 2014. The seroprevalence rate for the general population in 2015 (76% ± 5%) was higher than the initial estimate of 25% of chikungunya virus infections, which was based on the number of patients who sought medical care during the outbreak (*5*). The rate of asymptomatic chikungunya virus infections identified in this study (13% ± 2%) was comparable to rates reported by other countries (*10*).

Chikungunya virus antibody seroprevalence was much higher (76% ± 5%) than Zika virus antibody seroprevalence for the general population in 2015 (22% ± 6%) (*7*), despite the fact that both were virgin soil outbreaks occurring at the same place during 2 consecutive years (*5*,*6*). This discrepancy could be the result of distinct vector competence for Zika and chikungunya viruses in French Polynesia, as demonstrated in local mosquito species (*11*,*12*). Another possible explanation is that past exposure of the population to dengue viruses, as corroborated by the high level of anti-DENV neutralizing responses detected in samples collected in 2015, may have provided cross-protection against Zika virus infection (*13*). However, the occurrence of many cases of Guillain-Barré syndrome and Zika virus infection–associated congenital abnormalities (*8*,*14*) might also suggest that Zika virus immunopathogenesis is enhanced in the setting of high seroprevalence of DENV antibodies (*15*).
